# Statistical Accuracy in Rheumatology Research

**DOI:** 10.31138/mjr.30.4.207

**Published:** 2019-12-31

**Authors:** Ilke Coskun Benlidayi

**Affiliations:** Cukurova University Faculty of Medicine, Department of Physical Medicine and Rehabilitation, Adana, Turkey

**Keywords:** data accuracy, data analysis, data collection, data interpretation, rheumatology, medicine, statistics

## Abstract

Science and related research activities are always subject to errors. Statistical accuracy is necessary in order to overcome erroneous researching and reporting practices. The present article aimed to review the current literature on statistical errors in clinical medicine articles, and to provide rheumatologists with basic recommendations regarding the use of common statistical methods in research articles. With this purpose, PubMed/MEDLINE and Web of Science databases were searched by using relevant keywords. Data so far indicate that statistical errors are common in published articles from several disciplines of medicine. Statistics is the key element of any research activity, thus, implementing statistics at each step (hypothesis development, study design, sampling/data collection, data analysis, presentation) of every research is mandatory. In this regard, awareness of common statistical errors, basic knowledge on statistical methodology and consulting an expert in biostatistics from the beginning of the research process would be of value for rheumatologists.

## INTRODUCTION

Rheumatology is an ever-evolving area of medicine bearing a high research potential. Researching is necessary to build up evidence regarding the uncertain or unsolved topics in rheumatology. Publishing the results of these research activities is mandatory for delivering scientific knowledge among other researchers, health-care professionals, and patients. However, the accuracy, reliability and overall quality of publications are of great importance. Statistical accuracy is the key element for a reliable and quality research article. Therefore, it is of paramount importance to ascertain statistical appropriateness from the very beginning of a study until the end of the whole process. Science has always been subject to error.^1^ Publishing erroneous results derived from inadequately managed research projects may mislead future researchers as well as physicians who intend to use these results in daily clinical practice. In this regard, mentorship is of importance for guiding research naive trainees to manage research projects. A mentor is expected to assist a mentee on how to go about starting a research project, using search engines, discussing the whole process with a biostatistics expert and taking the project to completion. On the other hand, awareness about common errors in scientific research is also essential.

Over the past years, authors have focused on the importance of statistical correctness in medical research articles from different disciplines.^2–14^ However, data on statistical errors and/or accuracy in rheumatology articles are scarce.^12,15^ Given the lack in the literature, the present article aimed to provide recommendations for rheumatologists to ascertain statistical appropriateness in research papers by highlighting common statistical errors in clinical medicine articles.

## SEARCH STRATEGY

The present article followed the search strategy recommended for narrative reviews.^16^ Accordingly, PubMed/MEDLINE and Web of Science databases were searched by using the keywords “statistics”, “statistical analysis”, “errors”, “mistakes”, “rheumatology”, “medicine” in combination with appropriate logical connectors. Articles published during the last 5 years until 25^th^ January 2019 and those written in the English language were included in this narrative review. The exclusion criteria were as follows: i) letters to editors, ii) commentaries, iii) abstracts and iv) unpublished data. Following the literature search, retrieved articles were assessed for eligibility, and duplicate items or those unrelated to the topic were excluded. Remaining articles’ reference lists were checked, and relevant articles were further included in the narrative review.

## IMPLEMENTING STATISTICAL PERSPECTIVE AT EACH STEP OF A RESEARCH ARTICLE

**Figure 1.**
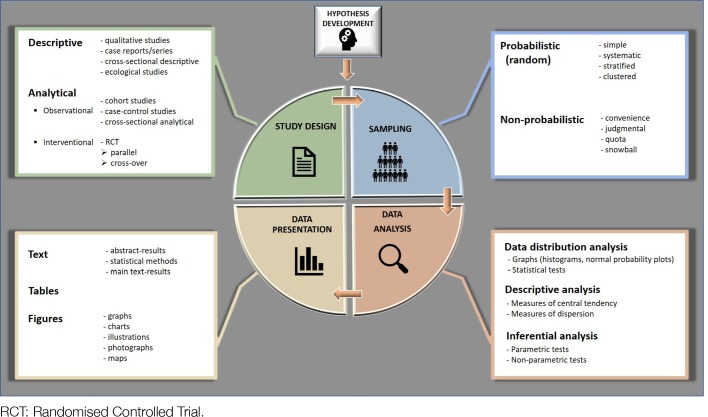
Research steps and statistical considerations.

**Figure 2.**
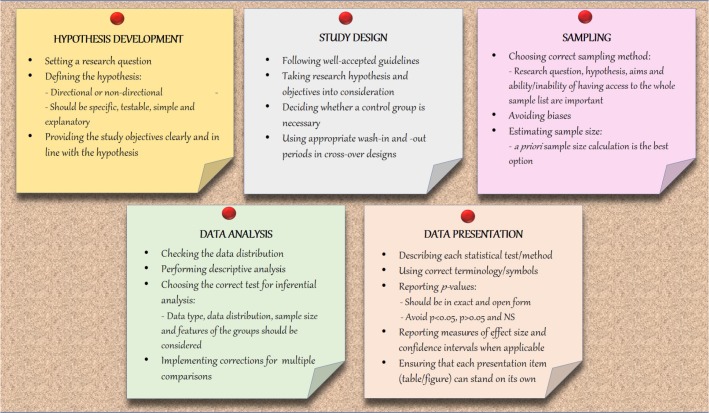
Tips for statistical accuracy in research articles.

It is of great importance to implement a statistical perspective at each step of any research.^17^ These steps can be summarised as research question/hypothesis development, study design, sampling/data collection, data analysis and data interpretation/presentation (*Figures 1 and 2*).

### Research Question and Hypothesis Development

Every study is designed around a research question. Thereafter, a hypothesis is set upon this question, as the hypothetico-deductive approach is followed by most medical scientific projects.^18^ A hypothesis is a testable statement created by the researchers. It is the working instrument of theory and relates theory to observation or vice versa. Hypotheses are relational propositions providing the researcher with a relational statement and a tentative explanation of phenomena.^1^

#### What are the features of a good hypothesis?

A good hypothesis must be specific and based on an appropriate research question. It must also be simple, with explanatory power. Most important of all, the developed hypothesis must be testable.^19^ After the testing procedure, researchers will either retain the null hypothesis (H_0_ or HN) or reject it in favour of the alternate hypothesis (H_1_ or HA).

The null hypothesis states that there is no accurate relation between tested variables. On the other hand, the alternate hypothesis states that H_0_ is not correct. Hypothesis testing is necessary to reject the null hypothesis or its alternative. Nevertheless, what should be kept in mind is that not rejecting H_0_ does not necessarily mean that the null hypothesis is true, as it might also indicate the insufficiency/deficiency of evidence against the null hypothesis.

The alternate hypothesis can be either directional or non-directional. The latter does not specify the direction of the relationship, whilst the directional hypothesis predicts a direction to the expected findings. At this step, what is already known from the past literature would guide the researchers in deciding the type of the alternative hypothesis.^19^

### Study Design

Research hypothesis should be tested on an accurate sample, and by using the correct study design. The design of a study can be either descriptive or analytical in nature; either observational or interventional in terms of methodology. Descriptive studies examine the distribution of one or more variables without providing any casualty. These are qualitative studies, case reports/series, cross-sectional descriptive studies (surveys) and ecological studies.^20,21^ On the other hand, analytical studies quantify the relationship between variables. Analytical studies can be designed either as observational (non-experimental) or interventional (experimental). Analytical observational studies observe the outcomes of any exposure without the active involvement of the researchers. Cohort studies, analytical cross-sectional studies, and case-control studies are examples of analytic observational research studies.^3,20^ On the other hand, interventional studies require the active involvement of the researchers.^20^ Randomised controlled trials are the prototypes of interventional studies.^3,20^ Two standard types of randomised controlled trials are the parallel and cross-over designed trials.^21^ Following well-accepted guidelines such as Consolidated Standards of Reporting Trials (CONSORT), the Strengthening the Reporting of Observational Studies in Epidemiology (STROBE), the Transparent Reporting of Evaluations with Nonrandomised Designs (TREND), and the Statistical Analysis and Methods in the Published Literature (SAMPL) is highly recommended to keep good research practice and standards in researching.^11,22–25^

#### Which design is the best for my study?

The research hypothesis and the related aims are major determinants while choosing the design of a study. If researchers target to examine the prevalence/incidence of a certain rheumatic disease or health-related characteristics of patients with this rheumatic disease, a descriptive study design should be set. On the other hand, if the aim is to analyse the effect of a factor (eg, smoking) on the disease-related variables of a rheumatic disease, the research design should be analytic.

Cross-sectional design can be used to determine the frequency of a disease. Moreover, it can also help to analyse the diagnostic procedures or disease-related parameters. However, if the prognostic value of any risk factor will be tested, the best design is the prospective cohort. On some occasions, the time required to observe this prognostic effect is too long. For these instances, retrospective cohort design can be applied. Nevertheless, in retrospectively designed studies, it is hard to eliminate or control the other confounders of disease prognosis.^27^ Randomised controlled trials should be the choice if the researchers aim to test the efficacy/effectiveness of any intervention. A parallel or cross-over design can be applied following the randomisation procedure. In a parallel designed trial comparing the efficacy of two biologics (A and B) in patients with ankylosing spondylitis; patients will be assigned either to receive biologic A or biologic B throughout the entire study period. On the other hand, in cross-over designs, patient groups will cross over from biologic A or biologic B during the course of the trial and vice versa. Since every individual serves as his/her control, cross-over design eliminates inter-individual differences and their potential effect on treatment outcomes. However, one important handicap of a cross-over design is the carry-over effect of each intervention. Appropriate use of wash-in and wash-out periods is essential in order to overcome this handicap.^21^ Pan et al., in their systematic review, appraised the methodological and reporting quality of randomised controlled trials conducted among patients with rheumatoid arthritis.^28^ They reported a high risk of bias and poor adherence to recommended reporting standards for randomised controlled trials.^28^ Randomised controlled trials stand as the hallmark of evidence-based medicine and it is of great importance to keep standards in sampling and randomisation while conducting these studies. However, it may be hard to apply randomisation in real-world practice. Therefore, “real” clinical premises and settings are also of value in providing complementary evidence.^29^

### Sampling and Data Collection

Sampling is the selection of a sample population (study population) from the whole population (target population). The study sample must represent the whole population properly in order to draw reliable conclusions regarding the target population by interpreting the results obtained from this sample. Flawed selection procedure causes a lack of representativeness. Therefore, sampling methodology should be set meticulously to achieve an accurate sample population.^30,31^

#### Which sampling method should I use for my study?

There are two main methods of sampling: i) probabilistic (random) sampling and ii) non-probabilistic sampling. Probabilistic sampling is the most recommended way of sampling, since this method almost ascertains the representation of the whole population by the sample population. Probabilistic sampling can be further categorised into simple, systematic, stratified and clustered random sampling.^31^ Simple random sampling refers to the randomisation of the whole population by using a random numbers table or a computer-based random list. On the other hand, systematic random sampling uses a systematic rule or a fixed interval for randomisation.^30^ Using these two sampling methods might have some limitations. If the researchers aim to include subjects from different age groups to their study, simple/systematic random sampling may not fully meet the researchers’ needs. In other words, simple sampling may fail to obtain adequate sampling from all age strata in the population. For these instances, stratified random sampling would work much better. Stratified random sampling is a modified simple sampling method, in which the division of the target population into strata is followed by random sampling from each stratum. The researchers divide the whole population into subgroups according to a factor such as age, gender, level of education or diagnosis.^30^ Then subjects are selected within each subgroup either through simple or systematic sampling.^31^ Research objectives and hypothesis are important while deciding which factor of division will be applied. If the researchers are not able to access the whole sample list due to the large size of the population, cluster sampling should be the way of choice.^30^ For example, if a nation-wide research is planned on knee osteoarthritis, the target population can be divided into clusters. Individual centres or general-practitioner practices can be assigned as clusters. Thereafter, other sampling methods are applied in order to select patients with knee osteoarthritis from each cluster/centre.^30^ This method can be regarded as an example of multi-stage sampling.^30^ If researchers plan to conduct a population-based observational study aiming to determine the risk factors of osteoarthritis, a multistage (complex) sampling will be appropriate.

In non-probabilistic sampling, the subjects are selected in a non-systematic process; thus, are usually not representatives of the target population. However, this method is still valuable in answering some research questions or generating new research hypotheses. Non-probabilistic sampling has several types including convenience (accidental), judgmental (purposive), quota and snowball sampling.^30,31^ The convenience sampling is the most widely used sampling method in clinical trials. In this method, subjects are selected according to their convenient accessibility. Thus, this method is subject to volunteer bias. Judgmental sampling selects subjects by the choice of the researchers and is subject to investigator bias.^30^ In quota sampling, quotas are chosen in order to represent the features of the target population and sampling units are selected to complete the quotas.^31^ Snowball sampling is used when a target sample cannot be located in a specific place. Each participant is asked to indicate other potential participants.^30,31^

#### How many subjects should I include in my study?

One of the major steps prior to data collection is the sample size estimation. Nour-Eldein reported that almost one-third of the family medicine articles assessed in the study did not mention sample size calculation.^11^ Sample size estimation enables the researchers to approximate the enough number of subjects to achieve acceptable study power-thus veracious results as the greater the power the lower the proportion of “false-negatives”.^32,33^ Running studies with low statistical power will reduce not only the likelihood of detecting true effects, but also the risk that a nominally statistically significant result (such as *p*<0.05) actually denotes a ‘real’ true effect.^32^ A large enough sample size is required to diminish the type-II error. Type-II error (β-error) refers to the wrong decision when a statistical test fails to reject a false H_0_. The Greek letter β represents the likelihood of making type-II error, that is to say, false-negative rate. For any sample, the maximum amount of false-negative results is desired as being 20%.^3^ Since the power of the study is expressed as 1-β, an experiment with a β of 0.20 will have a power of 80%.^34^ On the other hand, running studies with more than enough patients would increase the cost and put a needless number of patients at risk of potential harm.^15^ Running an *a priori* power analysis by taking significance level, power and effect size into consideration would be the best option to estimate the sample size of the planned study.^32,34^

### Data Analysis

Analysing the data is the most exciting step of research. Using correct statistical methods for data analysis is crucial in order to avoid incoherent, misleading and suboptimal results.

#### What is the way to accurately analyse data?

The first step of accurate data analysis is sound statistical design. If the researchers have performed the above-mentioned procedures including hypothesis development, study design, and sampling properly by taking the study aim and research question into consideration, it means that they are almost halfway through in statistical analysis. There are several statistical packages and numerous statistical tests for data analysis. Researchers should decide which statistical test is appropriate for the data.^35^ Bahar et al. evaluated 141 articles published in 6 cytopathology journals in 2014.^2^ The most common type of errors in the commission errors (misuse of statistical methods in reporting results) category was the failure to select appropriate statistical methods.^2^ Typical examples include using parametric tests for non-parametric data, running independent sample tests for paired data and Pearson’s chi-squared test for inappropriate sample size.^2^ Thus, the only possible way to decide the test to use is the recognition of the sample and related data. At that point, researchers should pay attention to data type, data distribution, sample size and dependent/independent groups/variables in the study.^35^

Data type is a major determinant of accurate data analysis. Allam et al. reported that statistical tests are incompatible with the type of data in more than half of the studies (53.2%) evaluated in their study.^35^ Variable type can be either qualitative or quantitative. Nominal and ordinal variables are representatives of quantitative data.^36^ Variables expressed by nominal or ordinal values are also regarded as categorical variables. For example, in an osteoarthritis study examining the relation of falls with disease severity, occupation is regarded as a nominal variable. On the other hand, Kellgren-Lawrence grade of osteoarthritis is an ordinal variable. Quantitative variables are numeric and subdivided to continuous and discrete variables. Discrete items are expressed by whole numbers, whereas continuous items can be recorded as any value.^36^ Regarding the above-mentioned study, the number of falls is discrete data, whilst body weight or height are continuous variables. The dependency/independency of each variable or tested groups is another important point while choosing the statistical test. The decision of running paired or unpaired tests are made according to this point. On the other hand, the decision of performing parametric or non-parametric tests is based upon the distribution of data.

#### Which statistical tests should I apply to my data?

Descriptive statistics is the first step in statistical analysis. It provides description about the measures of central tendency (mean, median, mode), as well as the measures of dispersion (standard deviation, variance, standard error of mean, range, percentiles).^36^ Mean is the arithmetic average of a certain variable and may be affected by the extreme variables/outliers. Median refers to the 50^th^ percentile, while the inter-percentile (inter-quartile) range represents the observations between the 75^th^ and 25^th^ percentile (the middle 50%) of the observations.^36^

Data distribution should be tested in order to decide which of the above-mentioned measures will be used to report the data.^14^ Testing data distribution is also required before moving to the second step-the inferential analysis. Data distribution is evaluated by statistical tests (ie, Kolmogorov-Smirnov and Shapiro-Wilks tests) and graphical analyses including histograms and normal probability plots. Histogram is a graphical technique on which the skewness and kurtosis of a data set can be evaluated. The standard normal distribution curve has a symmetrical bell shape.^3,14,36^ Skewness is the degree of distortion from this bell-shaped curve and regarded as the measure of asymmetry.^36^ Kurtosis is the degree of tailedness of a distribution. In addition to the graphical evaluation, it is of value to assess skewness and kurtosis values by using representative indexes as well.

Inferential statistics uses data of a sample population to make inferences in the larger collection of the population, and can be applied after testing the distribution of data. Parametric tests are applied for normally distributed quantitative data, whilst nonparametric tests are performed when the assumption of normality is not met.^36^ One of the most common misconceptions in data analysis is to regard nonparametric statistical tests as distribution/assumption-free or less powerful than the parametric tests. Instead, large-sample approximate tests are assumed to follow known distributions and, on some occasions, nonparametric tests can be more powerful than their parametric counterparts.^7^

If a comparative analysis is the case, it is important whether the groups are dependent or not.^36,37^ Parametric tests used to compare two paired groups and more than 2 matched groups are paired *t*-test and repeated measures analysis of variance (ANOVA), respectively. Nonparametric counterparts of these tests are Wilcoxon’s signed rank test and Friedman’s test. On the other hand, parametric tests used to compare two unpaired groups and more than 2 unmatched groups are unpaired *t*-test and ANOVA, respectively. Nonparametric alternatives of these tests are the Mann-Whitney *U* test and Kruskal-Wallis test.^2,36,38^ In case of multiple inferences on the same data sets or for comparisons of more than 2 groups, a post-hoc correction should be applied,^35^ since testing multiple hypotheses lead an inflated type-I error rate (false positivity), unless correction methods are used.^7^

Categorical data presented in frequencies/percentages are tested by using the Pearson’s chi-squared test, Fisher’s exact or McNemar’s tests, etc.^36^ If more than 20% of the cells have frequencies <5 (which indicates at least 5 observations in each category), Fisher’s exact test should be used instead of the Pearson’s chi-squared test.^5,11^ As for correlation studies, normally distributed data is tested by Pearson’s correlation analysis, whereas the Spearman’s correlation test is used for data beyond normal distribution.^2,36^

### Data Interpretation and Presentation

Data presentation is as important as data analysis in research. Providing the readers with proper presentations would allow accurate transmission of scientific evidence among researchers. Gunel Karadeniz et al. founded that 147/157 articles pusblished in radiology journals had at least one statistical error, and statistical error distribution did not differ between journals with impact factors ≥2 and <2.^9^ The most common type of error was incorrect data summarising (65.6%).^9^ In order to summarise the data correctly, researchers should understand and interpret the results accurately. On several occasions, *p*-values, which are definitely the most common statistical measure reported in articles are misinterpret by the authors.^7,12,39,40^

#### How should I interpret p-value results?

Significance is often discussed in terms of *p-*values (ranging from 0 to 1). On the basis of a prespecified significance level (α), *p*-values less than α indicates statistically significance in which the null hypothesis is rejected.^7^ The smaller the *p*-value, the greater the incompatibility of the data with H_0_.^41^ However, it does not imply that the difference is big or relevant.^42^ On the other hand, larger *p*-values suggest that there is insufficient evidence/information to reject H_0_. At that point, it is a mistake to focus solely on the results of null-hypothesis significance testing, that is to say, the *p*-value.^7,25,41^ Researchers should also pay attention to the magnitude of association-usually referred to as the effect size. The American Statistical Association’s statement on statistical significance and *p*-values indicates that a *p*-value, by itself, does not measure the size of effect or the importance of a finding.^7,41^ For a better interpretation of the results, it is of value to take measures of obtained effect sizes, minimal clinically important differences and confidence intervals into consideration, instead of relying solely on the *p*-values.^7,38,43^

#### How should I present my statistical output?

Satisfactory presentation of statistical output is an issue of concern for researchers. There are several ways for data presentation including texts, tables, and figures (graphs, charts, illustrations, photographs, and maps). Essentially, authors start presenting the study data as a text inside the abstract of their paper. At that point, being concise and explanatory is the key point. Firstly, a brief presentation of the sampling results such as sample size, age, gender, disease duration should be provided. Thereafter, the main finding of the study should be given. Results with regard to the secondary objective(s) may follow the main finding. Wherever possible/applicable, findings should be provided along with relevant *p*-values, correlation coefficients and confidence intervals inside the abstract.^44^ The second step should be the presentation of statistical methodology. Statistical methodology should be given at the end of the Methods section as a separate paragraph.^38^ Each statistical methods paragraph should be unique, as one “generic” paragraph would not fit all studies.^45^ Gunel Karadeniz et al. showed that almost 10% of the articles failed to provide statistical methodology.^9^ In this section of the article, data analysis technique(s), statistical software package used, accepted significance level should be provided. When multiple statistical tests or techniques are applied, the authors should state which test is used for which data, instead of using phrases such as “where applicable” or “where appropriate”.^3^ When multiple comparisons are performed, a multiple comparison correction/α level correction should be provided.^35^

The main arena for data presentation is the Results section of the paper. The Results text should be written after interpreting the data correctly, and by using appropriate terminology. Unfortunately, misinterpretation and misuse of statistical terminology/symbols are common in scientific articles.^35^ It is an issue of concern to focus on reporting positive statistical results belonging to secondary/tertiary aims in order to motivate the paper. However, regardless of the results being positive or negative, the greater focus should be given on the data related to the primary hypothesis.^45^ Researchers should also avoid selective reporting practices, as non-reported secondary findings and/or non-significant results can also be important and informative for readers, future researchers, overall for community and science.^6^

The results section should start with summarising each variable by descriptive statistics. Normally distributed variables are presented with mean and standard deviation, whereas skewed data are presented in median and range/interquartile range. It is of value to provide not only the size of the range but also the minimum and maximum values of ranges or upper and lower boundaries of interquartile ranges.^25^ The second step is the presentation of inferential statistical test results. At this step, authors should pay attention to present the results as much detailed as possible. For instance, instead of stating as “Minimum joint space width of the medial tibiofemoral joint differed significantly between patients with knee osteoarthritis and controls”, results should be expressed as “Minimum joint space width of the medial tibiofemoral joint was significantly narrower in patients with knee osteoarthritis than that in controls (p=0.02).^44^ Total sample and group sizes for each analysis should be provided.^25^ A common mistake while interpreting/presenting the results of randomised controlled trials or interventional studies is the use of within-group differences to infer between-group differences. For example, researchers may intend to evaluate the effect of aerobic training in fibromyalgia patients via a randomised controlled trial. The results may reveal that the experimental group showed significant improvement in outcome measures, while the controls showed no significant improvement over time. By solely using these within-group data, it will be a major mistake to conclude that aerobic training is effective in fibromyalgia.^45^ Rather, researchers should base their inference on the significance of the difference between groups.^7^ Gelman and Stern highlighted this important point in their article entitled “The difference between ‘significant’ and ‘not significant’ is not itself statistically significant”.^46^ Researchers should also avoid using the word “correlated” when only a comparative analysis was carried out without any correlation analysis.^2^

As for the interpretation of correlation analysis, researchers should not miss that a correlation itself does not necessarily represent a causal relationship; instead, it is regarded as one of the necessary conditions of causation.^10,42^ Therefore, researchers should avoid the word “predicted” when only a correlation analysis was performed.^2^ While reporting coefficients in association with correlation and regression, or reporting effect size measures such as absolute/relative risks, incidence/survival rates, odds/hazard ratios, confidence intervals (measure of precision) should be provided.^2,25^ Regression equation should be given for simple or multiple regression analysis.^25^ For multiple regression, variable selection process (eg, forward-stepwise) should be reported.^11,25^ As for diagnostic accuracy studies, the quantification of the statistical tests can be obtained by sensitivity, specificity, positive/negative predictive values, positive/negative likelihood ratios, odds ratio and/or the area under the receiver operating curve (ROC).

A big amount of errors in data presentation is related to *p*-values. Regarding the articles published in radiology journals, common mistakes related to *p*-values are: lack of reporting *p*-values, giving *p*-values in closed forms (ie, *p*<0.05 or *p*>0.05), incorrect expression of *p*-values (ie, *p*<0.0005 or *p*=0.000), providing *p*-values at conclusion section of the manuscript and using colon instead of equal sign in presentation (*p*:0.001 instead of *p*=0.001). It is recommended to describe *p*-values to 3 decimal places and exact values.^10^ On the other hand, it is of great value to provide the readers with the size of the effects studied. Reporting effect sizes or mean differences with confidence intervals would allow the readers to evaluate the power of the study and to clarify whether enough subjects were tested.^6,7^

Creating tables is an appropriate way to present large amounts of data. On the other hand, figure presentations such as graphs and charts are good at depicting the relation between series of numbers. Scatter plots are used to depict the association between two variables. Bar graphs are appropriate to indicate comparative results of different groups. They can be created horizontally or vertically, where the length (height) of the bar represents the amount of information. Pie charts are useful for representing data classified in different categories. Line plots are used to represent time-series data. Box and whisker charts are useful for presenting nonparametric data.^47^ Each table or graph should be self-explanatory and stand on its own regardless of the relevant text. Every presenting unit (graph, chart, table, figure) should have an accurate title. All abbreviations should be given in full at footnotes or figure legends. Authors should pay attention to referring each table or figure in the relevant part of the text.

## CONCLUSION

The guidance of statistics is required at each step of research. Implementing statistics would ensure adequate data collection, appropriate statistical analysis, and presentation. Statistical misuse is common among practices of medicine. Further research is required to better identify the misuse of statistical methods in rheumatology papers. It is of utmost importance for rheumatologists to be aware of the common errors in statistical use. Moreover, basic knowledge of statistical tools/techniques is essential. Supervision by an expert on biostatistics is recommended from the beginning of each research until the end of the whole process. In order to increase the statistical accuracy in scientific articles, it is useful to run a statistical review and/or provide the assigned reviewers with statistical checklists. Most importantly, basic education on statistical use and research methodology should be implemented in both under-graduate and post-graduate education in medicine.
